# Inter-day test–retest reliability and feasibility of isokinetic, isometric, and isotonic measurements to assess quadriceps endurance in people with chronic obstructive pulmonary disease: A multicenter study

**DOI:** 10.1177/1479973118816497

**Published:** 2018-12-17

**Authors:** Erik Frykholm, Sarah Géphine, Didier Saey, Hieronymus van Hees, Arthur Lemson, Peter Klijn, François Maltais, André Nyberg

**Affiliations:** 1 Department of Community Medicine and Rehabilitation, Unit of Physiotherapy, Umeå University, Umeå, Sweden; 2 Quebec Heart and Lung Institute Research Centre, Faculty of medicine, Laval University, Quebec city, Canada; 3 Department of Pulmonary Diseases, Radboud University Medical Center, Nijmegen, the Netherlands; 4 Department of Pulmonology, Pulmonary Rehabilitation Centre Merem, Hilversum, the Netherlands; 5 Department of Pulmonary Medicine, Amsterdam University Medical Center, Amsterdam, the Netherlands

**Keywords:** Intra-rater agreement, reproducibility, COPD, leg extension, lower limb muscle endurance

## Abstract

The aims were to determine reliability and feasibility of measurements to assess quadriceps endurance in people with chronic obstructive pulmonary disease. Sixty participants (forced expiratory volume in one second (mean ± standard deviation) 55 ± 18% of predicted, age 67 ± 8 years) were tested in an inter-day, test–retest design. Isokinetic, isometric, and isotonic protocols were performed using a computerized dynamometer. Test–retest relative and absolute reliability was determined via intraclass correlation coefficient (ICC), coefficient of variation (CV%), and limits of agreement (LoA%). Isokinetic total work demonstrated very high relative reliability (ICC: [95% confidence interval] = 0.98 [0.94–0.99]) and the best absolute reliability (CV% (LoA%) = 6.5% (18.0%)). Isokinetic fatigue index, isometric, and isotonic measures demonstrated low-to-high relative reliability (ICC = 0.64 [0.46–0.77], 0.88 [0.76–0.94], 0.91 [0.85–0.94]), and measures of absolute reliability (CV% (LoA%)) were 20.3% (56.4%), 14.9% (40.8%), and 15.8% (43.1%). For isokinetic total work and isometric measurements, participants performed better on retest (4.8% and 10.0%, respectively). The feasibility was similar across protocols with an average time consumption of less than 7.5 minutes. In conclusion, isokinetic, isometric, and isotonic measurements of quadriceps endurance were feasible to a similar extent and presented low-to-very high relative reliability. Absolute reliability seems to favor isokinetic total work measurements.

## Introduction

Impaired limb muscle function as evidenced by a reduction in strength^1^ and/or endurance^2^ of the quadriceps muscle is a common secondary consequence of chronic obstructive pulmonary disease (COPD), intimately associated with important clinical outcomes such as reduced quality of life, exercise intolerance, greater healthcare utilization, and premature mortality.^3^ A corollary of the prognostic and clinical importance of quadriceps function in COPD is that its assessment in clinical practice is highly recommended.^3,4^ The assessment could be performed using either static (isometric) or dynamic muscle contractions including isokinetic (constant pace) or isotonic (constant load) contractions.^4^ Isometric (sustained) contractions have been recommended for measuring quadriceps strength.^3,5^ Several protocols (including different measurements within isokinetic, isometric, and isotonic protocols) have been used to assess quadriceps endurance in the COPD population,^2^ but there is still no consensus on which protocol and outcome measure that is preferable in this context.^3^ Before the use of any protocol could become a reality in clinical practice several aspects, including but not limited to, the feasibility and reliability of measurement protocols needs to be determined. The reliability assessment should preferably include both relative and absolute reliability, where relative reliability is the degree to which individuals maintain their position in a sample with repeated assessments and absolute reliability is the degree to which repeated assessments vary for individuals.^6^ In people with COPD, one study has evaluated the reliability of an isokinetic protocol to asses quadriceps endurance using measurements of total work and fatigability (fatigue index) over 30 maximal repetitions.^7^ The authors report intraclass correlation coefficients (ICCs; relative reliability) of 0.93 and 0.92 and a minimal detectable change (absolute reliability) of 10% and 13%, respectively.^7^ Another study that compared quadriceps endurance between healthy subjects and people with COPD using an isotonic protocol, with measurements of time to exertion, reported high relative reliability (ICC > 0.74) for both groups.^8^ We could not find any previous report of the reliability of isometric quadriceps endurance protocols in people with COPD. However, in healthy adults, the relative reliability of an isometric quadriceps endurance protocol was high (ICC = 0.87).^9^ Neither could we identify any study investigating feasibility aspects of quadriceps muscle endurance assessments in people with COPD.

The primary aim of this study was, therefore, to determine the relative and absolute inter-day reliability of isokinetic, isometric and isotonic protocols for evaluating quadriceps endurance in people with COPD, including measures of isokinetic total work, isokinetic fatigue index, isometric time to exhaustion, and isotonic repetitions to exhaustion. A secondary aim was to examine the feasibility of conducting the three protocols. We hypothesized that the ICCs would be very high for isokinetic total work and isokinetic fatigue index (ICC > 0.90)^7^ and high for isometric time to exhaustion (ICC > 0.85)^9^ and isotonic repetitions to exhaustion (ICC > 0.70).^8^ In addition, we hypothesized that each protocol would be highly feasible, requiring less than 20 minutes for completion and being associated with little/no adverse events, apart from the discomfort associated with the procedure.

## Method

### Study design and participants

This was a multicenter inter-day reliability study with a test–retest design following the Guidelines for Reporting Reliability and Agreement Studies.^10^ The study was conducted at Umeå University, Umeå, Sweden; Université Laval, Québec city, Canada; and two pulmonary rehabilitation centers in the Netherlands: Radboud University medical center, Nijmegen, and Pulmonary Rehabilitation Centre Merem, Hilversum. Participants were enrolled at each center using a convenience sampling method if they were at least 40 years of age with a spirometrically confirmed diagnosis of mild to very severe COPD.^11^ Exclusion criteria consisted of recent COPD exacerbation (within the preceding 4 weeks); muscular-, rheumatic-, or cardiac disorders impacting testing procedures; and practicing regular exercise specifically aimed to strengthen the quadriceps. The study was approved by the respective local ethical boards (Umeå: DNR: 2015-426-31 M, 2016-379-32 M; Québec: CER: 21322; Arnhem/Nijmegen: CMO: NL59926.091.16) and all participants gave a written informed consent before the study commenced.

### Procedure

Each participant attended one inclusion visit followed by test and retest visits separated by 5–9 days. At the inclusion visit, the participants performed the COPD assessment test, the modified British medical research council questionnaire on breathlessness, the 6-minute walk test (6MWT), spirometry,^12^ and a quadriceps strength test (maximal voluntary contraction (MVC) test). Quadriceps strength was assessed isometrically with a computerized dynamometer (Biodex System Pro 3 or 4 (which can be used interchangeably), Biodex Medical Systems, Shirley, New York, USA) on the dominant leg stated by the participant. If there was uncertainty, the test was performed on the right leg. Participants were positioned with the knee placed at 90° angle, the center of movement at the knee joint as close as possible to the center of movement of the machine, and the lever arm firmly attached approximately 3 cm above the lateral malleolus. The arms were crossed over the chest and straps were used over the pelvis and thigh. The test consisted of five maximal isometric contractions of 5 seconds, separated by 1 minute of rest. Strong verbal encouragement was given to motivate maximal effort. Maximum torque in newton meter (Nm) was noted and the mean of the two highest reproducible (within 10% difference) contractions was used.^3^

### Quadriceps endurance measurements

**Figure 1.**
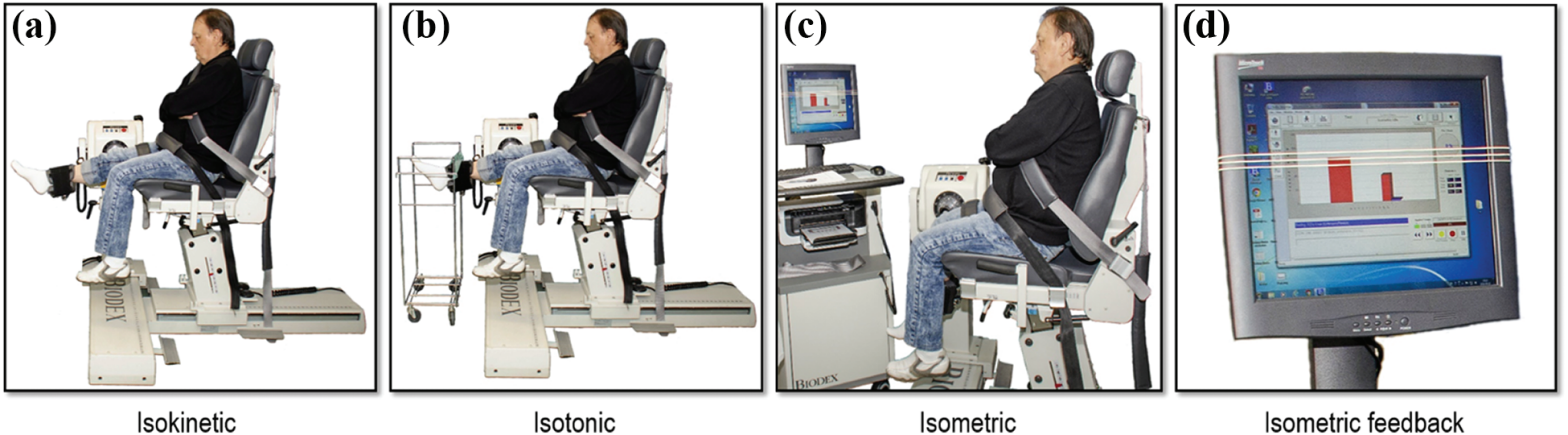
Setting for the isokinetic endurance test of 30 maximal repetitions at 90°/second (a), the isotonic endurance test of repetitions to exhaustion at 30% of the MVC (b), and the isometric test of time to exhaustion (c). For isokinetic (a) and isotonic (b) measurements, the range of movement in the knee joint was set from 90° to full extension (−5°), while for the isometric measurements (c), the knee was placed at 90° angle. During the isotonic test (b), a string was mounted on a movable construction to provide visual feedback on the desired ROM. During the isometric test, the display for visual feedback showed a bar of the applied force and three markers corresponding to 50, 55, and 65% of the participant’s MVC. The instruction was to keep the force between the 65% and 55% markers for as long as possible. The test was ended when the applied force fell below 50% MVC. Neutral feedback was given if the force dropped lower than 55% of MVC. MVC: maximal voluntary contraction; ROM: range of motion.

Test and retest visits included quadriceps endurance assessments using isokinetic, isometric, and isotonic protocols of leg extension, with each protocol performed once at test and once at retest (Figure 1(a) to (d)). The order of testing was randomized by a computer program and the same randomized order was used in both test and retest visits. Participants were positioned using the same settings as for the MVC test, for the dynamic tests the range of motion (ROM) was from 90° knee flexion to the individuals’ maximal extension −5°.^13^ All protocols included a standardized warm-up specific to the test being performed: 5 isokinetic contractions with progressing force (last contraction maximal), 3 sustained 5-second isometric contractions (separated by 30 second rest) at 20%, 40%, and 60% of MVC, and 10 isotonic repetitions at 15% of MVC. The specific warm-up was followed by 2 minutes of rest, and each test was separated by 30 minutes of rest. For the assessment of feasibility, the revised category-ratio 0–10 scale (Borg CR10)^14^ was used before and after each test for ratings of dyspnea and leg fatigue; the total time from start of instructions to postexercise Borg CR10 ratings was noted as the execution time, and any adverse event was noted and described.

Test of isokinetic endurance was performed using a protocol consisting of 30 maximal contractions at a pace of 90°/second. During the test, strong verbal encouragement was given on every contraction. Measurements were total work reported in joule and a fatigue index based on the difference in work performed during the first and last third of the test.^7^ The isometric endurance test protocol was performed by asking participants to maintain, for as long as possible, an isometric quadriceps contraction representing 60% of the individual isometric MVC force. The participants had visual feedback from a computer screen showing their applied force, described further in Figure 1. The test was ended if the force of the contraction was less than 50% of the MVC for three consecutive seconds. Measurement of time (in seconds) during which the contraction was maintained higher than 50% of the MVC was used for analysis.^15^ The isotonic endurance test protocol was performed by doing as many repetitions as possible using a resistance of 30% of isometric MVC maintaining an externally set pace by a metronome at 60 beats per minute (i.e. 30 repetitions/minute). The test was ended when the participant, in three consecutive repetitions, failed to reach full ROM, or failed to maintain the accurate pace. The ROM was individualized for each patient; a string was mounted on a movable construction to provide visual feedback on the desired ROM (see Figure 1). Neutral feedback was given concerning ROM and pace. Number of repetitions performed was used as measurement of endurance.^8^

### Sample size calculation and statistical analysis

Fifty-one participants assessed two times were needed to detect an ICC with the lower limit of the 95% confidence interval (CI) > 0.70 for the isotonic test, according to Wolak’s estimation of sample sizes for reliability analyses.^16^ Bland–Altman plots were used to visualize original test–retest data using reference lines at the mean difference (MD) and at 1.96 standard deviation (SD) of the MD, known as the limits of agreement (LoA).^17^ Heteroscedasticity was evaluated by plots of the absolute difference to the mean and by a Kendall’s Tau test for correlation. Heteroscedasticity was present for three variables (isokinetic total work, isometric, and isotonic measurements, range *r* = 0.251–0.371, *p* < 0.05). Since the heteroscedasticity was reduced (and were no longer significant) after base-10 logarithmic transformation (range *r* = 0.033–0.086, *p* > 0.05), the logarithmic values were used for further analyses of these variables.^6,18^ Normal distribution of the differences between test and retest was then confirmed by a Shapiro–Wilk test with *p* > 0.05.

Test–retest relative reliability was assessed using an ICC 3.1 procedure presented with 95% CI and was proceeded by a paired *t* test to test for systematic bias.^19^ Munro’s descriptors were applied to the lower limit of the 95% CI^20^: very low = 0.15–0.24, low = 0.25–0.49, moderate = 0.50–0.69, high = 0.70–0.89, and very high = 0.90–1.00.^21^ To describe test–retest absolute reliability for logarithmic transformed variables, the coefficient of variation (CV%) and 95% LoA% were used as described by Euser et al.^22^ Where the CV% estimates the precision of a single measure, and the LoA% estimates the minimum amount of change that could be considered a true change between repeated measurements, both expressed in percent of individual mean values. Briefly, the mean within-individual standard deviation (wSD), calculated as the square root of the mean within-individual variance, was used to present CV% and LoA% using the formulas:

CV%=ln(10)×wSD×100

$$\text{LoA}\%=2(10^{a}-1)/(10^{a}+1)\times 100$$

where $\;a=1.96\times \sqrt{2}\times \text{wSD}$

For fatigue index, whose distribution was homoscedastic, CV% and LoA% were calculated using the formulas:

CV%=wSD/grand mean×100

$$\text{LoA}\%=1.96\times \sqrt{2}\times \text{wSD}/\text{grand mean}\times 100$$

The feasibility aspects (mean values of dyspnea; leg fatigue; and time to perform the isokinetic, isometric, and isotonic protocols) were tested for differences between the three protocols using a repeated measures analysis of variance, with post hoc analyses using Bonferroni correction for multiple comparisons. Dyspnea and leg fatigue were also compared using paired sample *t* tests within each protocol. Occurrence of adverse events was descriptively reported.

The Statistical Package for the Social Sciences (version 24, IBM SPSS Statistics, Armonk, New York, USA) was used with a 5% *α* level for statistical significance set a priori.

## Results

**Figure 2.**
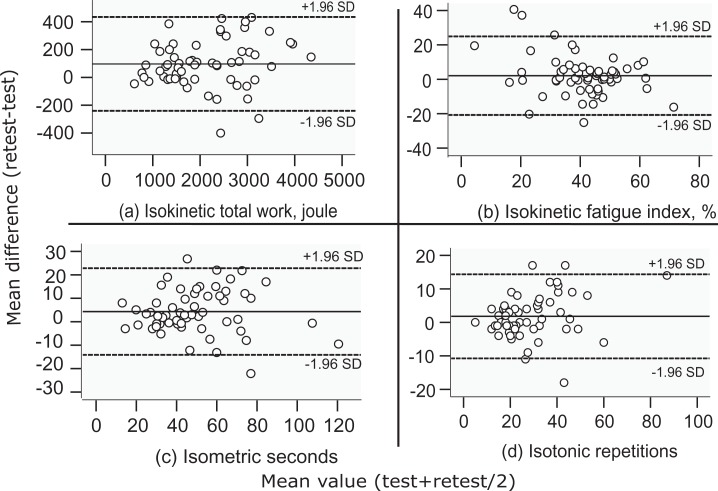
Bland–Altman plots of individual variation between test–retest measurements using the original data for isokinetic total work in joule (a), isokinetic fatigue index calculated as the ratio of the decline in work during the last 10 repetitions to the first 10 repetitions of the total 30 maximal isokinetic repetitions (b), isometric time in seconds to exhaustion above 50% of MVC (c), and isotonic repetitions to exhaustion at 30% of MVC (d) measurement results. Mean values of the test and retest measurements are displayed on the *x*-axis and the difference between retest and test on the *y*-axis (positive values indicates a higher result on retest). Reference lines at the mean difference and LoA (mean difference ± 1.96 SD of the mean difference). MVC: maximal voluntary contraction; LoA: limits of agreement; SD: standard deviation.

Characteristics of study participants are shown in Table 1. Bland–Altman plots of the original data are shown in Figure 2(a) to (d), visualizing the heteroscedasticity (i.e. the amount of error increased as the measured values increased) that was present for three variables (isokinetic total work, isometric, and isotonic measurements).

**Table 1.** Participant characteristics.^a^

*N*	60
Age (years)	67 ± 8
Body mass index	26.6 ± 5.0
Male/female	36/24 (60/40)
FEV_1_ (L)	1.5 ± 0.6
FEV_1_ (percentage of predicted)	55 ± 18
FVC (L)	3.4 ± 0.9
FVC (percentage of predicted)	99 ± 22
FEV_1_/FVC (%)	44 ± 13
mMRC	
0	1 (1.7)
1	24 (40.0)
2	11 (18.3)
3	6 (10.0)
4	18 (30.0)
CAT (1–40)	17 ± 7
6MWT (m)	453 ± 116
6MWT (percentage of predicted)	81 ± 21
Isometric MVC (Nm)	130 ± 45
Isometric MVC (percentage of predicted)	76 ± 22

FVC: forced vital capacity; FEV_1_: forced expiratory volume in one second; mMRC: modified medical research council dyspnea scale, higher values indicates more dyspnea; CAT: COPD assessment test, higher values indicates higher impact of COPD; 6MWT: 6-minute walk test; MVC: maximal voluntary contraction; SD: standard deviation.

^a^ Values are shown as the mean ± SD or category *n* (%).

Table 2 presents mean values and reliability aspects of original and log-transformed data of the test and retest occasions. Isokinetic total work demonstrated very high test–retest relative reliability (ICC: [95% CI] = 0.98 [0.94 to 0.99]), isokinetic fatigue index low relative reliability (0.64 [0.46 to 0.77]), while the isometric time to exhaustion and isotonic repetitions to exhaustion demonstrated high relative reliability (0.88 [0.76 to 0.94] and 0.91 [0.85 to 0.94], respectively). The best absolute reliability was found for isokinetic total work (CV% (LoA%) = 6.5% (18.0%)), whereas the other measurements presented higher absolute reliability (CV% (LoA%) range 14.9–20.3% (40.8–56.4%)). The participants performed significantly better on retest in measurements of isokinetic total work and isometric time to exhaustion (differences of 4.8% and 10.0%, respectively, both *p* < 0.001).

**Table 2.** Reliability results of the four endurance measurements.^a^

		Original data				Log10-transformed data				
	*N*	Test	Retest	MD	LoA	MD%	*P*	CV%	LoA%	ICC [95% CI]
Isokinetic joules	60	2078 ± 876	2174 ± 906	96	327	4.8	<0.001	6.5	18.0	0.98 [0.94, 0.99]
Isokinetic fatigue index %	60	39.8 ± 15.4	41.9 ± 11.9	2.1	22.9	5.1^b^	0.17^b^	20.3^b^	56.4^b^	0.64 [0.46, 0.77]^b^
Isometric seconds	58^c^	47 ± 22	52 ± 21	4.3	18.5	10.0	<0.001	15.8	43.1	0.88 [0.76, 0.94]
Isotonic repetitions	59^d^	27 ± 13	29 ± 15	2	12	5.1	0.06	14.9	40.8	0.91 [0.85, 0.94]

MD: mean difference, difference between test and retest positive values indicating higher values at retest; CI: confidence interval; LoA: limits of agreement, smallest difference to be considered real with 95% CI; MD%: relative mean difference between test and retest; CV%: coefficient of variation; LoA%: smallest difference to be considered real with 95% CI; ICC: intraclass correlation coefficient (3.1); SD: standard deviation.

^a^ Values are shown as the mean ± SD or 95% CI.

^b^ Original data used for all analyses.

^c^ Two participants excluded as knee pain/discomfort affected test performance.

^d^ One participant excluded due to technical issues.

The feasibility of the measurement protocols is shown in Table 3. In brief, the average time to perform either of the three endurance protocols was less than 7.5 minutes. Leg fatigue was consistently rated higher than dyspnea (*p* < 0.001 for all comparisons), irrespective of protocol. Dyspnea was rated highest in the isokinetic protocol (both comparisons *p* < 0.001). In addition, three patients reported pain (in the knee) during the isometric protocol, two of which terminated one or both test occasions due to pain and were therefore excluded from the reliability analyses. One participant was excluded from the isotonic protocol due to technical issues. No other adverse event was reported.

**Table 3.** Feasibility results of the three endurance protocols.^a^

	*N*	Time (minute:second)	Dyspnea	Leg fatigue
Isokinetic	60	6:16 ± 1:14^b^	3.4 ± 1.7^c^	4.5 ± 1.7^d^
Isometric	58^e^	7:05 ± 1:35	2.5 ± 1.5	4.1 ± 1.9^d,f^
Isotonic	59^g^	7:17 ± 1:29	2.7 ± 1.6	4.6 ± 1.8^d^

SD: standard deviation.

^a^ Values shown are means and SD; execution time measured in seconds including instructions, warm-up, rest, test, and Borg ratings; dyspnea and leg fatigue rated on Borg CR10.

^b^ The isokinetic protocol took less time than the isometric and isotonic protocols, for both comparisons *p* < 0.001.

^c^ The mean rating of dyspnea was higher in the isokinetic protocol than in the isometric and isotonic protocols, for both comparisons *p* < 0.001.

^d^ Leg fatigue was rated higher than dyspnea for each protocol, *p* < 0.001 for all comparisons.

^e^ Two participants excluded as knee pain/discomfort affected test performance.

^f^ The mean rating of leg fatigue was lower in the isometric protocol compared to the isotonic protocol *p* = 0.01.

^g^ One participant missing due to technical issues.

## Discussion

To the best of our knowledge, this is the first study to investigate the reliability properties of different protocols and measurements to assess quadriceps endurance in COPD. Overall, measures obtained by isokinetic, isometric, and isotonic protocols present high to very high relative reliability with the exception of measurements of isokinetic fatigue index that present low relative reliability. CV% ranged from 6.5% to 20.3%. The three protocols display similar feasibility with minor differences in perception of symptoms, time to complete the measurements, and adverse events.

The relative reliability results from the present study are consistent with earlier studies^5,7,9^ and our hypothesis for isokinetic total work (very high), isometric time to exhaustion (high), and isotonic repetitions to exhaustion (high). However, relative reliability for the isokinetic fatigue index was lower than previously reported for a protocol using 30 repetitions at a speed of 90°/second but higher than a protocol using 180°/second.^7^ This could be due to difficulties to follow the instructions of performing maximal effort on each isokinetic contraction. Indeed, three participants displayed none to a very slight decline over time on the test occasion (−5.3% to 1.8%), indicating a failure of the protocol to cause fatigue. Excluding these three participants affect both the relative and absolute reliability of the measurement (ICC = 0.70, 95% CI [0.54–0.81], CV% = 15.5, LoA% = 42.8). Also, at the test occasion, the group range was −5.3 to 79.5, and at the retest occasion it was 12.7–66.4, indicating group regression toward the mean. A familiarization session, modification of instructions, and/or modification on the calculation of the fatigue index could possibly improve reliability. The absolute reliability of the isokinetic measurement total work (CV% = 6.5%) was substantially better than for the other protocols and measurements (CV% range = 14.9–20.3%). In addition, the LoA% indicated the smallest detectable change for the measurement total work of the isokinetic protocol approximated around 20%, while for the other protocols and measurements it was above 40%. These observed differences in absolute reliability might affect the applicability of the measurements to clinical trials and practice—a lower absolute reliability indicates that the measurement is more sensitive to true changes in performance. However, this needs to be further evaluated by investigating the measurements responsiveness to change and determining the minimal clinically important change. For example, in a recent review, the magnitude of improvement of limb muscle endurance seemed to differ depending on the measurement used.^23^ In that review, the mean improvement was 16% following isokinetic total work endurance measurements, while corresponding values were 42% for isometric and 71% for isotonic endurance protocols. This would indicate that even though the measures of absolute reliability seem to differ between protocols and measurements, this does not automatically mean that the ability of detecting changes is different.

Participants performed better on retest during isokinetic total work and isometric measurements (by 4.8% and 10.0%, respectively, *p* < 0.001). This could be due to a learning effect in performing the tests. A familiarization session and/or performing additional tests at each visit would most likely reduce the possible learning effect.^7,24^

The isokinetic, isometric, and isotonic endurance protocols display similar feasibility; the average time needed to complete the measurements was less than 7.5 minutes for each protocol. This can be compared to the 6MWT, the gold standard for measuring functional capacity in people with COPD, which takes a minimum of 16 minutes including instructions and rest, to complete.^25^ The actual time for each test, excluding instructions, was for the majority of participants similar to the 1 minute sit to stand test.^26^ Indicating that the test may measure similar aspects of functional capacity. How these tests are related should be further investigated. Regarding perceived symptoms, the participants rated leg fatigue higher than dyspnea. We interpret this information as a validation that the participants perceived performance limited by quadriceps function and not by cardiopulmonary function, although we acknowledge that cardiopulmonary responses during the tests were not assessed. During the isometric protocol, two participants stopped because of knee pain/discomfort and one additional participant reported knee pain/discomfort post assessment. These were judged as minor adverse events, considering that these participants could continue with other quadriceps muscle endurance tests after the 30-minute rest or could walk unaffected if it was the last endurance protocol. Noticeably, neither of these participants reported knee pain during the dynamic tests.

One strength of the present study was the multicenter design that provided a diverse sample of people with COPD with sufficient power for narrow 95% CI; another was that heteroscedasticity was examined and the analyses adjusted accordingly, that is, data were logarithmic transformed where appropriate before further analyses. Providing the result from logarithmic transformed variables increases the transferability of result to the heterogenic population of people with COPD by providing a relative measure of absolute reliability that can be applied at an individual level. Indeed, the individual mean value of isokinetic total work during 30 repetitions in the study was between 613 and 4345 joules, and the absolute difference between 0 and 431 joules (significantly associated (*r* = 0.321, *p* < 0.001)), indicating that the measurement error increases with greater mean values and should, therefore, be expressed relative to the mean.

However, even though logarithmic transformation is recommended when addressing heteroscedastic data,^6,18^ it reduces the ability to report results in common units for absolute reliability such as standard error of the measurement and minimal detectable change on the original scale.^6^ In addition, the criteria used for ending isotonic test (the test was ended when the participant, in three consecutive reps, failed to reach full ROM or failed to maintain the accurate pace) leave room for interpretation. Thus, the test–retest reliability results of this trial are valid only if the same assessor is used at test and retest occasions. The inter-rater agreement could not be assessed due to the multicenter design of the study but should be a goal of future trials. Furthermore, to improve usability in clinical settings and reduce the burden on participants, the present study did not use a familiarization session, and only one test at each test occasion was performed. But since there was a significant systematic bias in two measurements, the lack of additional tests/familiarization session reduces our ability to draw firm conclusions on how test procedures should be designed to maximize reliability for the individual patient. Furthermore, even though an isokinetic protocol measuring total work seems to be the most reliable to measure quadriceps endurance, the protocol demands a computerized dynamometer whose costs and limited availability may impede its use in clinical settings.^4^ The present study used a computerized dynamometer for all protocols, and this was done to ensure identical positioning of the participants across protocols and involved recruitment sites. The isometric and isotonic protocols could, however, be used with other equipment. For example, the isometric protocol can be performed with a cheaper strain-gauge system or a fixed hand dynamometer.^5,27^ Nevertheless, further research is needed to evaluate the protocols and measurements construct validity, strategies to reduce the systematic bias, and responsiveness to change.

## Conclusion

In the assessment of quadriceps endurance among people with COPD, measures obtained by isokinetic, isometric, and isotonic protocols present high to very high relative reliability with the exception of measurements of isokinetic fatigue index that present low relative reliability. CV% ranged from 6.5% to 20.3%. A familiarization session/test might be needed before actual assessment is performed due to changes in performance over time. The three protocols display similar feasibility with acceptable time consumption, limited perceived dyspnea compared to leg fatigue, and no major adverse advents.
